# The Impact of the Test Dissociation on the Binocular Balance of Children

**DOI:** 10.3390/clinpract13040088

**Published:** 2023-08-18

**Authors:** Danjela Ibrahimi, Marcos Aviles, Juvenal Rodríguez-Reséndiz

**Affiliations:** 1 Faculty of Engineering, Autonomous University of Querétaro, Santiago de Querétaro 76010, Mexico; marcosaviles@ieee.org; 2 Faculty of Medicine, Autonomous University of Querétaro, Santiago de Querétaro 76176, Mexico

**Keywords:** Howell test, alternate Cover test, Thorington test, dissociated phoria state, break value of near point of convergence, degree of stereopsis

## Abstract

Purpose: this research compared the dissociated phoria at near and distance fixation in free space using the Howell test, alternate Cover test, and Thorington test. Methods: 220 healthy Mexican children (mean age 8.3±2.5 years) participated in this study. Phorias were quantified at both distances using each test, from the least to the most disruptive. The stereopsis degree and near point of convergence (break/recovery) were analyzed to understand their role in the visual system’s sensorimotor balance. Results: statistically significant differences were found among techniques, with a higher congruence for the EF. However, only the Howell and Thorington tests can be interchanged. The break value and near exophoria relate to each other and affect the stereopsis degree, whereas age is associated with the stereopsis degree and break value. Conclusions: the three techniques cannot be interchanged except for the Howell and Thorington test for the EF at far. The differences in the mode of dissociation could relate to the results.

## 1. Introduction

The visual system constructs a mental representation of the world around us and contributes to our ability to navigate physical space and interact with the environment. The visual system takes the lead and becomes the dominant sensory modality when the child begins to stand. From this point on, planned motor movements are mostly visually guided, and the need to explore in depth the visual performance of our children and its further development becomes crucial [1].

In daily clinical practice, apart from visual acuity, developmental optometrists emphasize the analysis of the accommodation and phoria state, vergences, and stereopsis degree, among others, as being essential components of the binocular system [2]. The techniques used to evaluate these visual system elements are categorized from more to less disruptive and provide quantifiable data to diagnose and propose an accurate treatment when necessary.

This paper aims to evaluate the phoria state of children at near and distance fixation in free space through three different techniques, to understand the impact of the test dissociation on their binocular response. The phoria state describes the misalignment of the visual axes elicited when fusion is artificially disrupted by using an occluder, the Maddox rod, or a vertical prism. Commonly, two visual conditions can be found during the evaluation of the phoria state of a patient: a high or a low tonic vergence, known as esophoria and exophoria [3,4]. The binocular state can be affected by the ability of the visual system to compensate for the magnitude of exophoria or esophoria measured during the evaluation by interrupting the sensorimotor balance and its performance [5].

Our ability to perceive the three-dimensional space, ocular movements, vergence flexibility, relationship between the accommodation and vergence system, visual–motor integration skills, etc., can be affected by the phoria state [6]. High magnitude of phorias can result in tropias (strabismus, such as exo or esotropia), being diplopia the first symptom of the decompensated phoria state [7,8]. Double vision occurs as the visual cortex cannot immediately suppress the deviated eye’s image, which results in constant suppression, as the best visual–cortical adaptation to avoid diplopia [9]. Research suggests that phoria levels can change throughout the day, depending on the amount of effort on a sustained visual task, and that the cerebellar cortex mediates the phoria adaptation and some aspects of learning [10]. Therefore, its assessment becomes critical, even more in children, where neural plasticity allows rapid visual–cortical adaptations, and their neurological development is influenced by the environment and the context they live in [11]. In the field of visual health professionals, different clinical techniques exist to quantify the magnitude of the phoria in free space at distance and near. This research compares the dissociated phoria state at near and distance fixation in free space, using three different techniques: the Howell test, alternate Cover test, and Thorington test. Each technique presents a different degree of dissociation, with the Howell test as the least disruptive test [4]. To evaluate the horizontal phoria, a vertical prism is used for the Howell test to disrupt binocularity, a translucent occluder is needed for the alternate Cover test, and a red Maddox rod is used for the Thorington test. The magnitude of phorias at near and distance were compared using parametric and non-parametric tests through the alternate Cover test, Howell test, and Thorington test to find their differences and similarities. Previous studies on the comparison of near-dissociated phoria state in free space exist, being that these evaluations were mostly performed in adults [12,13,14]. It is the first time that the phoria state from a large sample of Mexican children (220 participants, mean age 8.3 ± 2.5 years) and their binocular state is examined and compared to other ethnicities, generating new scientific data. The Thorington test, which has been compared to the alternate Cover test, von Graefe technique, and Maddox test, is not interchangeable with any of them, and any relationships found were from medium to low [13,14].

Furthermore, our research included a complete visual efficacy exam of each participant, and data about the degree of stereopsis, break, and recovery of the near point of convergence, visual acuity at distance and close, refractive status, and flat fusion at both distances were also considered. A detailed statistical analysis of the visual performance of participants was carried out to understand the interaction among the components of the visual system and their effect on its sensorimotor balance during childhood.

## 2. Methodology

This research compared the dissociated phoria state at near and distance fixations in free space, using three different techniques: the Howell test, alternate Cover test, and Thorington test. A total of 220 healthy patients from Querétaro, México, participated in this study: 132 males (60%) and 88 females (40%), mean age of 8.3 ± 2.5 years. Patients with strabismus, amblyopia, ocular pathologies, neurological conditions, Central Nervous System disorders (CNS), and premature and under medication were excluded from the study. The absence of strabismus was confirmed with the unilateral Cover test. The visual efficacy exam was performed at Brain Vision & Learning Center, a diagnostic center for visual problems, collaborating with the Autonomous University of Querétaro (UAQ), located at Boulevard Jurica la Campana, 1194, Juriquilla, Querétaro, México. Dr. Danjela Ibrahimi, professor-researcher of the Faculty of Engineering, UAQ, collected data. The number of patients was calculated based on the number of participants who met the inclusion criteria and decided to participate. The study was conducted according to the guidelines of the Declaration of Helsinki, and all parents signed the informed consent form before starting any procedure.

Inclusion criteria:

(i) Age 5.06 to 17.11;
(ii) VA ≥ 0.3 logMar;
(iii) No previous visual treatments;
(iv) No symptoms of visual distress.

Children from 5.06 to 17.11 were included to encompass all childhood (from the moment they start the reading and writing process, which happens during pre-first, to the point they become adults), which provides a clearer image of their phoria state and binocularity during this period of life. Moreover, the collaboration of children that already read and follow instructions is much better than that of younger children.

Ages were calculated based on their birth date and the evaluation date as follows:

- Evaluation date: 12 June 2023;
- Birth date: 10 May 2016;
- Chronological age: 02-01-07 (rounded to 7 years, 1 month = 7.01).

The visual efficacy evaluation was performed from 08:00 am to 12.00 am, after 8–10 h of sleep based on the age of participants, which allows the visual system to be more effective and provides reliable data. Visual exams were performed in the morning to avoid the visual system’s tiredness. The visual exam is at most 45 min and is adapted to the collaboration of each participant.

### 2.1. The Evaluation Followed Three Steps (One Step per Day)

**Step one:** detailed personal medical history of the patient and their family background.
**Step two:** determine the best optical prescription under the cycloplegic effect of 1% tropicamide [15], and subjective refraction afterwards.
**Step three:** evaluate the visual efficacy with the new prescription using the following motor and sensorial tests:
  (1) Visual acuity at 40 cm and 3 m using Bailey–Lovely charts (logMar).
  (2) Stereopsis at 40 cm using the Random-Dot 2 test, which goes from 500 (gross) to 12.5 (fine) seconds of arc.
  (3) Howell test at 33 cm and 3 m using the Howell test phoria card and a six-base down prism in front of the right eye.
  (4) Alternate Cover Test using a translucid occluder: at near fixation (40 cm) using a 20/30 single letter on the Gulden fixation stick, and at a distance (3 m), by isolating a 20/30 letter on the distance visual acuity chart.
  (5) Thorington test at 40 cm and 3 m holding a Maddox rod (with its horizontal axis) in front of the right eye and the penlight against the back of the card.
  (6) Worth dot test at 33 cm and 3 m, respectively, to evaluate flat fusion and suppression.
  (7) Near point of convergence test (NPC) and its recovery, using an accommodative target (four repetitions in total), and its mean value was recorded as the final one.
  (8) Monocular, estimated method retinoscopy at 40 cm (MEM), using the appropriate card based on the age and grade level of the patient.

As stated above, the phoria state at a distance and near fixation was determined using three different techniques in free space. The same examiner performed all tests, Dr. Danjela Ibrahimi, a specialist in vision and child development. The phoria state is measured in prismatic diopters, where a higher value describes a worse phoria state.

### 2.2. Howell Test

This is the least dissociative test. Two cards measured the phoria state at a distance and near fixation under normal light conditions (purchased by Bernell Corporation). The patient held a vertical prism of 6 dpt in front of the right eye, which provoked double vision. The participant reported the number aimed by the arrow, which represented its horizontal phoria. Direction and magnitude were recorded.

### 2.3. Alternate Cover Test

The doctor and patient sat in front of each other. To avoid the influence of proprioception on the result, the near fixation stick was held by the doctor’s assistant. At the same time, the patient was asked to keep the 20/30 letter on the stick clear while the doctor performed the alternate Cover test. To avoid deep binocular dissociation, a translucent occluder was used. Neutralization of the eye movement was reached using a prism bar in front of the right eye of the patient. The prism value and the base direction were recorded. For the phoria state at distance fixation, a 20/30 letter was isolated on the distance acuity visual chart, and the same procedure at near fixation was followed.

### 2.4. Thorington Test

This is the most dissociative test. To measure the phoria state at a distance and near fixation, the Bernell muscle imbalance measure cards were used (for distance and near) under normal light conditions. Therefore, the obtained results correspond to the natural binocular state of participants. The participant held a Maddox rod (with its horizontal axis) in front of the right eye while the doctor placed the penlight in the central small hole of the card. The participant reported the number on the card through which the red streak appeared to pass, and these values represented the horizontal phoria in prism diopters. The direction and the magnitude of the phoria were then recorded for both distances.

The reason to evaluate phorias in a fixed and not randomized order was to not compromise our data, as the more dissociative a test, the more dissociation is provoked in the visual system. Therefore, starting with the Howell test and finishing with the Thorington test avoids this effect. The motor and sensorial tests used in this research have already been standardized and accepted by the optometry and ophthalmology scientific community. They can be found described in detail in [4]. The evaluation was performed respecting all steps indicated in the above-mentioned book.

### 2.5. Statistical Analysis

The magnitude of phorias at near and distance were compared using parametric and non-parametric tests through the alternate Cover, Howell, and Thorington tests. The statistical analysis was performed using the SPSS Statistics Base 25.0 program. The normality of data distribution was checked with the Shapiro–Wilk (S–W) test. The confidence level (CI) used in this study was 95%, with α = 0.05. The Independent Samples *t*-test was conducted to analyze the means of two independent groups to determine their statistical differences when data were normally distributed, and the Mann–Whitney test was for the non-normally distributed data. The One-Way ANOVA for repeated measures with Bonferroni adjustments for multiple comparisons was used to compare the results obtained using the three different techniques for normally distributed data.

In contrast, the Friedman test with pairwise comparison was preferred when data were non-normally distributed. The Bland–Altman plot was performed to analyze the agreement between two different techniques. The regression analysis was performed when the relationship between one dependent and one or more independent variables was analyzed, and the Pearson correlation was used to estimate the power of a specific correlation.

## 3. Results

From the total 220 patients, 132 were males (60%) and 88 were females (40%), where 181 had exophoria (82.3%) and 39 presented esophoria (17.7%). A total of 91 patients were hyperopic (41.4%) and 21 myopic (9.6%) with or without astigmatism, 11 patients had pure astigmatism (5%), whereas 97 patients did not have any refractive error (44%) A total of 214 participants had flat fusion (evaluated using the Worth dot test) at distance fixation (97.3%). Only 6 patients presented diplopia (2.7%), while at near fixation, flat fusion was presented in 199 participants (90.5%); 20 had diplopia (9%), and only 1 child showed suppression of one eye (0.5%).

### 3.1. Descriptive Statistics and Analysis Based on Gender

Gender did not relate to visual acuity or stereopsis. Nor did the number of phorias measured through the alternate Cover, Howell, and Thorington tests at near and distance fixation show statistically significant differences between male and female participants. Differences in the refractive status of patients were only significant for hyperopia, with girls more hyperopic than boys. The independent sample *t*-test was used to analyze differences between groups based on gender, except for the esophoria state, myopia, and astigmatism, where the analysis was performed using the Mann–Whitney test considering the small sample and the non-normal data distribution analyzed by the Shapiro–Wilk test. Table 1 illustrates the mean value, standard deviation (Std), and *p*-value of the analyzed variables based on gender. The break and recovery of the NPC is not included in this table, as it is analyzed separately.

### 3.2. Comparison of the Magnitude of Phorias at Near and Distance, Measured through the Alternate Cover Test, Howell and Thorington Test

Considering the data distribution analyzed by Shapiro–Wilk, the One-Way ANOVA for repeated measures with Bonferroni for multiple comparisons was used to compare the degree of XF at a distance and near, whereas the Friedman test was usedfor EF. Table 2 presents the statistical analysis results.

At near fixation, the One-Way ANOVA for repeated measures showed that sphericity assumed (Mauchly´s test of sphericity >0.5), the test of within-subjects effects was statistically significant, F(2)=302.53, p<0.001 and Eta-squared =0.63. The within-subject contrasts and multivariate tests obtained the same statistically significant results. Likewise, statistically significant data were found at distance fixation. The within-subjects effects analysis showed F=44.23, p<0.001 and Eta-squared =0.20. Data were statistically significant for the within-subject contrasts and multivariate tests. These results indicate that the values obtained by each test differ significantly among them.

The Friedman test for the degree of EF showed statistically significant results at near and distance fixation, where for df=2, p<0.001 at both distances, while $\chi^{2}=60.50$ (near) and 15.63 (far), respectively. Posteriorly, The Wilcoxon signed-rank test was performed to determine where the differences lie. These results can be seen in Table 2(b).

Statistically significant differences were obtained from each test used, as seen in Table 2, except for the esophoria state at far when measured through the Howell and Thorington tests. The highest phoria values were obtained by the alternate Cover test, whereas the lowest ones by the Howell test, being that these differences were more noticeable at near fixation. The alternate Cover and Howell tests found the most significant difference in the magnitude of exophoria measured at near fixation. A minor difference, however, was seen in the magnitude of esophoria at distance fixation as defined by the Howell and Thorington test.

### 3.3. Bland–Altman Plot to Analyze the Agreement between the Used Techniques

**Figure 1:**
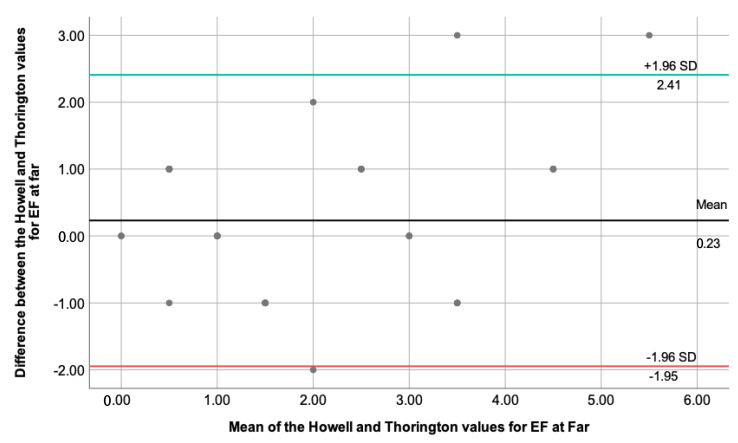
Depicts the Bland–Altman plot for the degree of esophoria at far, measured using the Howell and Thorington techniques. The BLACK line represents the mean value (0.23), while the green and red lines show the upper (2.41) and lower (−1.95) 95% CI, respectively (±1.96 standard deviations). Few outliers can be observed, whereas most data fit into ±1.96 standard deviations.

This section performed an extended statistical analysis, comparing two techniques each time. The difference between the two measures and the mean value of the two measures were necessary to perform the One-Sample *t*-test and see the necessity to build a Bland–Altman plot. Only for the degree of esophoria at far measured through the Howell and Thorington test, the One-Sample *t*-test showed a p=0.202, with t=1.29. The 95% CI was calculated using the mean (0.23) and standard deviation value (1.11), and the results are represented by Figure 1.

By looking at the plot, we are looking for evidence of proportional bias, and the regression analysis was performed to clarify this point. The linear regression analysis showed F(1)=2.89, unstandardized β=0.21, t=1.69 and p=0.098. The closer the unstandardized β value for the mean is to zero and the p>0.05, it is assumed that there is no proportional bias for the results obtained.

The One-Sample *t*-test showed p<0.001 for every two techniques compared, except for the degree of XF at far measured using the Howell and Thorington test (p=0.004) and EF at far measured through the alternate Cover and Thorington test (p=0.001). However, being p<0.05, their differences are statistically significant, and the Bland–Altman plot is not recommended.

### 3.4. Regression Analysis to Determine Variables That Could Predict the Degree of Stereopsis

The regression analysis was performed to determine which variables could predict the degree of stereopsis. A new variable was introduced in this phase, the break of NPC, related to patients’ vergence state. From the performed analysis, only the exophoria state at near and the break of NPC were related to the stereopsis value. The esophoric state was not related to the degree of stereopsis. The predictive models for every phoria test and the NPC break are presented in Table 3.

Table 3 shows that the strongest predictive variable for the stereopsis degree is the break of NPC (higher standardized beta-value and F-value than the rest), followed by the near exophoria state measured through the Howell test, alternate Cover test, and Thorington test as the last one.

**Figure 2:**
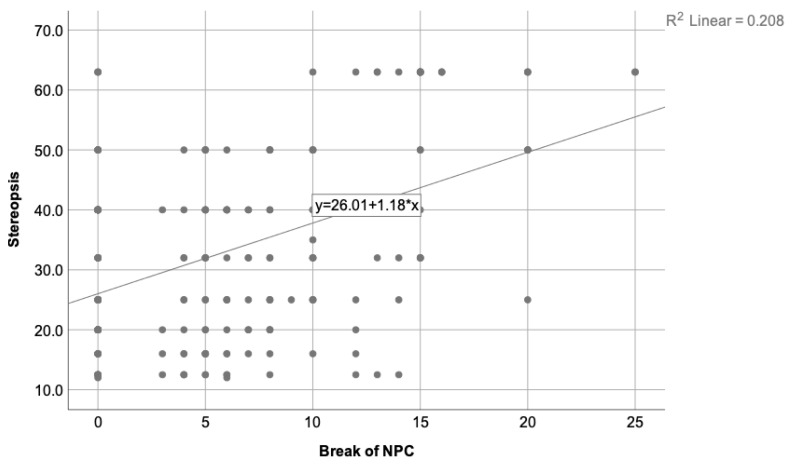
Depicts the correlation found between the stereopsis degree and the break of NPC, where $R^{2}$ = 0.208. A decreasing trend on the stereopsis degree is observed when the NPC value increases. Therefore, worse stereopsis is expected in patients with higher NPC value.

**Figure 3:**
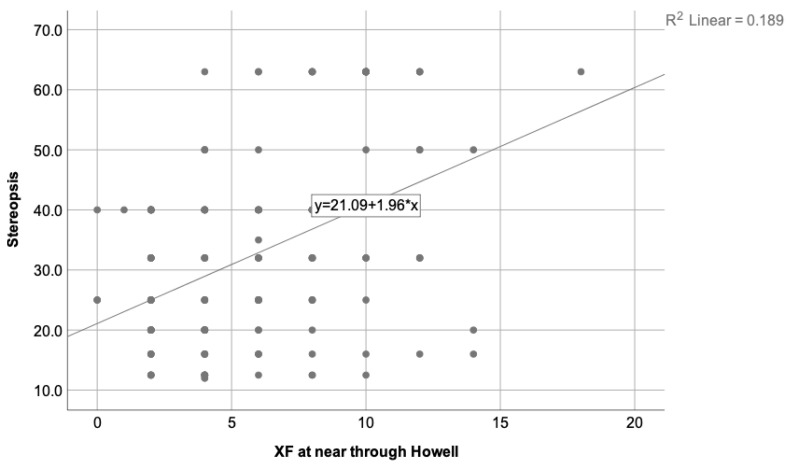
Illustrates the correlation found between the stereopsis degree and the exophoria state at near measured using the Howell test, where $R^{2}$ = 0.189. As the exophoria at near increases, the stereopsis decreases. Therefore, worse stereopsis is expected in patients with higher exophoria at near distances.

Figure 2 depicts the correlation found between the stereopsis degree and the break of NPC, while Figure 3 illustrates the correlation found between the stereopsis and the exophoria at near when the Howell test was used.

**Figure 4:**
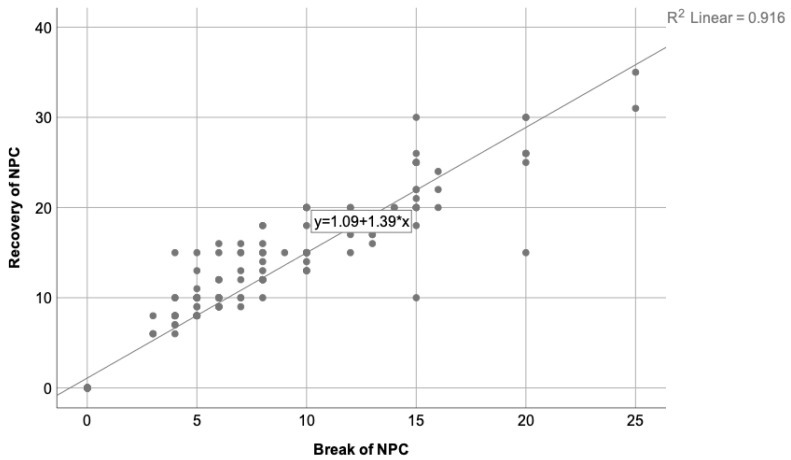
Represents the correlation state between the break and recovery of NPC, where $R^{2}$ = 0.916. As is to be expected, participants with a high break of NPC present higher recovery value as well, being that their values are proportional to each other.

The relationship between the break of NPC and its recovery was also analyzed, where the Pearson correlation coefficient was 0.96 and $R^{2}$ = 0.916. This means that break and recovery are proportional to each other, as it is expected to be. Figure 4 shows this correlation.

### 3.5. Statistical Analysis of the Stereopsis Degree, Phoria State and the Break of NPC Based on Age

Considering that the visual system changes during childhood, age is considered an important variable related to its performance. Therefore, three age groups were created to analyze the phoria state, stereopsis, and break of NPC, as follows: first group, 5.06 to 8.00 (62 patients, 28.2%); second group, 8.01 to 11.00 (111 patients, 50.5%); and third group, 11.01 to 17.11 (47 patients, 21.4%). The One-Way Anova Test was used to analyze and compare the means among the three groups. The Levene test of homogeneity of variances showed *p*-values greater than 0.05, suggesting that variances among groups were not significantly different. The Tuckey HSD was used for multiple comparisons. Results from the analysis of stereopsis and break of NPC are presented in Table 4.

Significant differences can be appreciated for the stereopsis degree when the first group (5.06–8.00 years) is compared to the other two groups. The most significant difference was found between the first (5.06-8.00 years) and the second group (8.01–11.00 years). No statistical differences were found between the second (8.01–11.00 years) and the third group (11.01–17.11 years). A higher stereopsis degree is measured in the second group (8.01–11.00 years) and lower in the first (5.06–8.00 years).

For the break value of NPC, the second group obtained the lowest value and the third the highest one. Statistically significant differences can be appreciated when the second group (8.01–11.00 years) is compared to the first (5.06–8.00 years) and third (11.01–17.11 years). The most significant difference was found between the mean break value of the second (8.01–11.00 years) and the third group (11.01–17.11 years). No differences were presented between the first (5.06–8.00 years) and the third group (11.01–17.11 years).

Regarding the phoria state, age was not related to its magnitude. More specifically, taking the alternate Cover test as a reference (the most used technique in the clinical practice of optometrists and ophthalmologists to evaluate the phoria state), p=0.19 and 0.11 for the XF value at distance and near fixation, whereas for EF, p=0.51 and 0.92 for distance and near respectively.

### 3.6. Statistical Analysis of the Stereopsis Degree and Phoria State Based on the Break Value of NPC

The break of NPC is associated with the exophoria state and the vergence system of an individual. Likewise, the exophoria can impact the participant’s binocular state, such as the stereopsis degree. Consequently, the break of NPC was divided into three groups, as follows: the first group included all participants with a break from 0–5 cm (122 patients, 55.5%); the second group was composed by participants with a break from 6–10 cm (55 patients, 25%), and the third one had a break from 11–25 cm (43 patients, 19.5%). The phoria state and stereopsis degree was analyzed based on the break value of the NPC. Break values within the 10 cm range are considered the norm.

**Figure 5:**
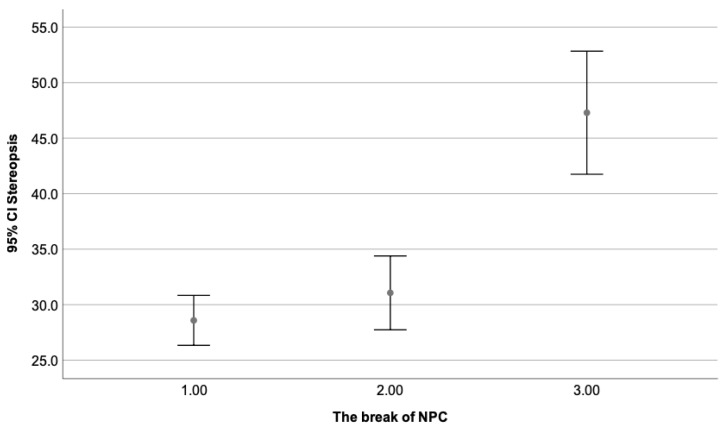
Shows the mean stereopsis degree as a dependent variable of the break value of NPC, where the third group (break of 11–25 cm) obtained the lowest value and the first one (break of 0–5 cm), the highest one. Statistically significant differences can be appreciated when the third group (11–25 cm) is compared to the first (0–5 cm) and second (6–10 cm). As expected, no differences were seen between the first (0–5 cm) and second group (6–10 cm), with values from 0–10 cm considered within normal limits.

As can be seen, by Table 5, significant differences were found when the third group (break of NPC 11–25 cm) was compared to the first and second one. The break value is considered within normal limits when it does not exceed the 10 cm range. Therefore, patients with a break beyond it present a diminished degree of stereopsis. No statistically significant differences were found when group one and two were compared, as both have a break value within normal ranges. Figure 5 illustrates the mean stereopsis degree as a dependent variable of the break value of NPC, where the third group (break of 11–25 cm) obtained the lowest value and the first one (break of 0–5 cm), the highest one.

**Figure 6:**
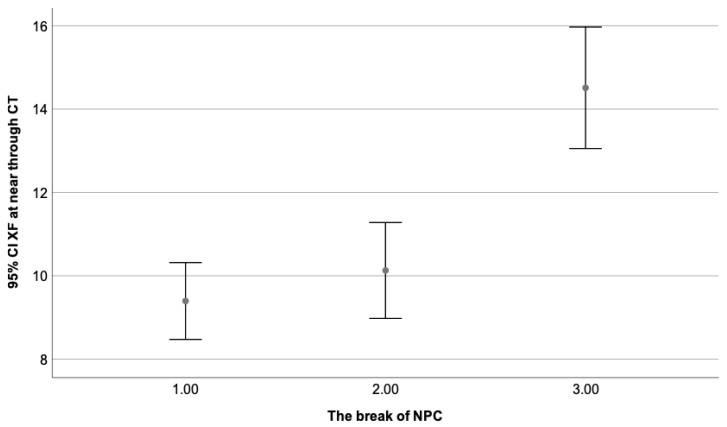
Presents the exophoria state at near as a dependent variable of the break value of NPC, where the third group (break of 11–25 cm) obtained the highest value and the first one (break of 0–5 cm), the lowest one. Statistically significant differences can be appreciated when the third group (11–25 cm) is compared to the first (0–5 cm) and second (6–10 cm). No differences were found between the first (0–5 cm) and the second group (6–10 cm). These values were considered within normal limits.

A total of 181 participants presented exophoria at near and distance fixation. From the total, 91 participants (50.3%) formed the first group (break of 0–5 cm), 47 participants (26%) formed the second group (break of 6–10 cm), and 43 participants (23.7%) were included in the third group (break of 11–25 cm). Results from the One-Way Anova with Tuckey HSD for multiple comparisons analysis are illustrated in Table 6. Patients with esophoria could not be analyzed, and considering the small sample, divided into groups, could not be representative. Figure 6 represents the mean value and Std of the exophoria state at near fixation only (as presenting a stronger correlation) as a dependent variable of the break value of NPC. A higher break value (worse) is related to a higher magnitude of exophoria (worse) at distance and near fixation.

## 4. Discussion

The phoria state, which describes the latent deviation of the visual axes, provoked when binocularity is disrupted, and both eyes are no longer collaborating [16], was analyzed in this research. The control of phorias as well as their adaptation levels, is a cortical phenomenon [17,18], and frequent changes in its magnitude can be seen during the day because of sustained fixation and high visual attention [17,18]. Therefore, the analysis of heterophorias becomes necessary in childhood. Many studies have been conducted on the reliability and comparison between different measurement techniques in past decades [19,20]. However, we focused on the most recent studies, knowing that with the development of technology, the learning conditions have changed, and the demand for close-distance work has increased. Consequently, the accommodative–vergence responses since childhood adapt to this new order, and their consequences should be reflected in the binocular state of children.

Even though recent studies on heterophorias have been conducted in adults and other ethnicities [12,13,14], it is the first time that such an analysis has been performed in children, covering a wide range of ages. This research used three techniques: the Howell test, the alternate Cover test, and the Thorington test. The Cover test is the most used technique among ophthalmologists and optometrists. It provides reasonable inter-examiner agreement [20]. However, the modified Thorington test is considered the best choice for phoria measurement because of the simplicity of its administration, as well as its repeatability [14]. On the other hand, the Howell test, considered the least disruptive one, has yet to be used and analyzed by visual health professionals [21]. The most recent studies published on the analysis of near heterophorias have been conducted in adults (18–80 years), while our sample consisted of children.

Secondly, it is the first time that these three techniques are compared among them and used to analyze the phoria state at distance fixation as well. Previous studies compared the near phoria measured with the trial frame with the conventional muscle balance card method and the conventional Maddox rod [13]. A second one compared the modified Thorington with the Maddox rod, Cover test, and von Graefe technique [13], a third one performed the same techniques as in [14] but focused on the within-session repeatability and normative data of these techniques, and a last one focused on comparing the Howell test with the modified Thorington method and the von Graefe technique [21]. There is a study on the heterophoria values in a small sample of children from 3 to 5 years compared to adults (19–28 years), but here, the MCS Power Refractor (eye tracker) and Cover test were used instead [22]. Another critical point of our data analysis is the interaction among essential components of the visual system and their impact on the binocularity of our children. The statistical analysis showed that most of our participants were exophoric (181 out of 220); similar observations were seen in previous studies [12,13].

Significant differences were found in the obtained values with the alternate Cover and Thorington test, and a possible reason for these results could be due to the different methods used for dissociation. Thorington test uses image distortion, while the occlusion dissociation method in the alternate Cover test could cause a deeper breakdown of fusional vergence, which may result in higher values compared to other techniques, as stated by previous researchers [12,13,14]. Likewise, the phoria quantification method should also be considered. Prism manipulation is required for the alternate Cover test, while scales are used for the Thorington test. Similar findings are reported in the adult population [20,21]. The Bland–Altman plot showed a good agreement between the Howell and Thorington test for the esophoria state at distance fixation, with a difference of less than two prismatic diopters, which is clinically accepted [23]. A previous study on the Howell and Thorington phoria tests showed that there was significantly less variability in interexaminer differences and obtained values between these two techniques when compared to the von Graefe method [21], which suggests that we should expect similar results when using them. Points to consider here are the test distance (the Howell test at near was presented at 33 cm while Thorington at 40 cm) and methods of dissociation (the Howell test uses prism doubling while Thorington image distortion), as well as the sample. Additionally, the visual system of children is still malleable, and more variation during their clinical examination could be observed as the case in point. A higher value variability was observed when the Howell and alternate Cover tests were compared. The dissociation method, the test distance, and the phoria quantification used by these techniques are different, which could explain these results. Another point to consider when comparing these techniques is that the alternate Cover test is a more objective technique, as the examiner determines the magnitude of the deviation without any verbal participation of the patients. The patient is instructed to look at an accommodative point while the examiner neutralizes the movement of the eyes with the bar prism. What is mainly required here is visual attention.

On the contrary, the Thorington and Howell tests are more subjective techniques, where the verbal collaboration of the patient is essential. The patient needs to give a verbal answer about what his visual system sees. Even though the sample was carefully used to have a good understanding and the tests are easy to administer, this aspect should always be considered during the exam evaluation. An interesting observation when paring the techniques to build the Bland–Altman plot is that less difference (however statistically significant) was found for the magnitude of XF at far measured using the Howell and Thorington test, and EF at far as measured through the alternate Cover and Thorington test. Therefore, a tendency was found for the EF and XF at far using the Howell and Thorington tests, and a higher congruence among the three techniques for the EF at far. The above-mentioned studies have focused on the near phoria condition of participants, and the distance phoria state has not been raised in any of them, so we consider that the level of accommodation could affect these results. It is already known that the level of accommodation generated during a habitual near task is higher than at a distance, so higher differences are expected to be found to be found at near than far [24].

The important question here would be which one is better than the other, or which one the examiner should rely on. To answer this question, it is crucial to review the patient’s medical history and relate the clinical findings to the signs and symptoms he reports. As a clinician, the closer the technique is to the natural binocular state of the patient, the most reliable the gathered data are. When we break fusion by using dissociative techniques to quantify the phoria state of the patient, the gathered data inform the examiner about the fragility of the visual system, its adaptability to the changes on the levels of sustained attention, and its flexibility [6]. When the repeatability between measures is high and consistent under different levels of dissociation, the visual system is considered stable and balanced. An easily dissociable visual system is fragile, where a high magnitude of phoria, often followed by accommodative and vergence dysfunction, can no longer be compensated [25]. It has been found that proximal cues can significantly contribute to the vergence system [26], and patients may rely on the knowledge of object distance for instrument accommodation rather than on the optical distance of the object [27]. Therefore, evaluating under conditions similar to the real day-to-day situation is recommended. One last point to consider in this research is age and its effect on the phoria state of the children. A previous cross-sectional study on the values of distance heterophoria and fusional ranges reported that the mean heterophoria value for distance fixation was exophoric (no effects of age observed), except in older participants [28]. In this paper, similar results were obtained, where most participants were exophoric at both distances, and age was unrelated to its magnitude. Likewise, children from 3 to 5 years old have shown similar heterophoria values at near and distance fixation when compared to adults [22].

The second part of the statistical analysis focused on the interaction among variables such as the stereopsis degree, magnitude/direction of phorias, and break/recovery of the near point of convergence to predict participants’ binocularity levels. The neurophysiology of the visual system explains that patients with binocular dysfunctions, such as convergence insufficiency (high break values and recovery of NPC), accommodative problems, vergence flexibility, etc., are followed by diminished stereopsis and poorer visual performance [2]. Our results showed that the break of NPC and the exophoria state at near fixation are the only predictors for the degree of stereopsis, where worse stereopsis (lower values) was found among children with a higher magnitude of exophoria. Likewise, participants with higher break values (worse break values) presented a higher magnitude of exophoria at near fixation (worse exophoria state). Additionally, age was related to the break value of NPC and stereopsis degree. Participants of the second group had the best stereopsis degree and the lowest break value of NPC. Our hypothesis here is that younger children (participants of the first group) could have less control over their visual system, and older ones (third group) may suffer from visual stress as the levels of sustained visual attention and near work tasks increase with age, affecting posture, ocular movement control, and components of the binocular system, obeying to the neurophysiology orders [29,30]. These results confirm what has been written in theory, that the visual system components interact among them and affect each other [31]. As researchers, we understand that every study can be followed by limitations, which we must minimize as much as possible. One limitation of this research could be the physiological changes in the individual related to attention and tiredness, which we tried to control with a good night’s sleep (8–10 h before the evaluation) and choosing morning hours for the visual exam (8–12 a.m.), where the visual system is more efficient and the collaboration of the participants is considered the best. Secondly, the accommodation targets can affect the phoria results. However, we tried to control this factor by giving proper instructions, following the standardized procedures, having good illumination, and performing all tests with the best optical prescription. Finally, we tried minimizing all probable potential biases as all measurements and data followed standardized procedures [4]. This research focused on the effect of test dissociation on the binocular response of children from early childhood to adulthood and the interaction of essential visual system components related to binocularity. The comparison of data from different countries and continents creates a more extensive and precise panorama of the impact of different ways of evaluation from early childhood to adulthood.

## 5. Conclusions

This research compared the dissociated phoria state at near and distance fixation in free space using three different techniques: the Howell test, the alternate Cover test, and the Thorington test. A total of 220 healthy Mexican children from Querétaro, 5.06 to 17.11 years, participated in this study. The degree of stereopsis, break, and recovery value of the near point of convergence was also analyzed to understand the interaction among them and their impact on the sensorimotor balance of the visual system. Most of our participants were exophoric and hyperopic. Statistically significant differences were found among techniques. No effects of age were observed. Only the Howell and Thorington test for the EF at far can be interchanged. Fewer differences among techniques have been found for the EF at far. Stereopsis is related to the exophoria at near and the break of NPC. Gender does not relate to any variable, whereas age is associated with stereopsis and the break of NPC. A better knowledge of the visual responses under different conditions and their interaction is the key to the assertive diagnosis and treatment program.

## Figures and Tables

**Table 1:** Descriptive statistics of the analyzed variables based on gender, where mean values, Std, and *p*-values are presented. The independent sample *t*-test was used for most of them except for the esophoria state, myopia, and astigmatism, where the analysis was performed using the Mann–Whitney test considering the small sample and the non-normal data distribution analyzed by the Shapiro–Wilk test.

	Boys	Girls	*p*-Value	Total
**Variables**	**Mean ± Std**	**Mean ± Std**		**Mean ± Std**
Age	7.90 ± 2.03	8.90 ± 3.08	0.006	8.30 ± 2.48
VA OD F	0.04 ± 0.07	0.04 ± 0.08	0.941	0.04 ± 0.07
VA OS F	0.04 ± 0.08	0.03 ± 0.07	0.467	0.04 ± 0.07
VA OD N	0.05 ± 0.07	0.06 ± 0.08	0.281	0.05 ± 0.08
VA OS N	0.06 ± 0.09	0.05 ± 0.08	0.685	0.06 ± 0.09
Stereopsis	32.36 ± 14.82	33.61 ± 16.39	0.565	32.86 ± 15.45
EF F CT/H/TH	2.65±2.55	3.38 ± 3.07	0.135	2.95 ± 2.76
	1.96 ± 1.64	2.13 ± 1.75	0.417	2.03 ± 1.64
	1.78 ± 1.23	1.88 ± 1.63	0.071	1.82 ± 1.37
EF N CT/H/TH	8.74 ± 4.11	9.25 ± 3.92	0.637	8.95 ± 3.99
	5.75 ± 3.17	5.38 ± 3.03	0.740	5.60 ± 3.08
	7.09 ± 2.95	7.56 ± 2.78	0.407	7.28 ± 2.86
XF F CT/H/TH	2.11 ± 2.59	2.11 ± 2.62	0.523	2.11 ± 2.60
	1.14 ± 1.47	1.01 ± 1.46	0.783	1.09 ± 1.46
	1.39 ± 1.44	1.26 ± 1.36	0.265	1.34 ± 1.41
XF N CT/H/TH	10.81 ± 5.04	10.79 ± 4.54	0.571	10.80 ± 4.80
	5.76 ± 3.44	5.49 ± 3.34	0.926	5.60 ± 3.39
	8.50 ± 3.97	8.08 ± 3.65	0.741	8.34 ± 3.84
Stereopsis	32.36 ± 14.82	33.61 ± 16.4	0.557	32.86 ± 15.45
Myopia OD	−1.48 ± 0.96	−2.56 ± 2.12	0.226	−1.89 ± 1.56
Myopia OS	−1.52 ± 0.93	−2.25 ± 1.76	0.378	−1.80 ± 1.32
Hyperopia OD	0.88 ± 0.43	1.39 ± 1.61	0.035	1.12 ± 1.17
Hyperopia OS	0.84 ± 0.43	1.49 ± 1.77	0.017	1.15 ± 1.29
Astigmatism OD	−0.86 ± 1.44	−1.45 ± 1.29	0.143	−1.15 ± 1.39
Astigmatism OS	−0.98 ± 1.29	−1.58 ± 1.41	0.204	−1.29 ± 1.38

VA, visual acuity; OD, oculus dexter; OS, oculus sinister; F, far; N, near; EF, esophoria; XF, exophoria; CT, Cover test; H, Howell test; TH, Thorington test; Std, standard deviation.

**Table 2:** (**a**) Phoria tests comparison using the One-Way ANOVA for repeated measures for XF at far and near fixation, where the *p*-value reflects the differences between them. Confidence Intervals are presented for a better understanding of the relationship between the analyzed variables. (**b**) Phoria tests comparison using the Wilcoxon signed-rank for EF at far and near fixation, where the *z*-value and *p*_np-value reflect the differences between them.

(**a**)			
Pairwise comparisons			
XF at Near			
		95% Confidence Interval for Difference	
**Tests compared**	p **-value**	**Lower Bound**	**Upper Bound**
Cover Test/Howell	<0.001	4.663	5.746
Cover Test/Thorington	<0.001	1.977	2.907
Thorington/Howell	<0.001	2.238	3.287
XF at Far			
Cover Test/Howell	<0.001	0.728	1.316
Cover Test/Thorington	<0.001	0.460	1.064
Thorington/Howell	0.012	0.045	0.474
(**b**)			
Pairwise comparisons			
EF at Far			
**Tests compared**		z **-value**	***p* _np-value**
Cover Test/Howell		3.14	0.002
Cover Test/Thorington		3.88	<0.001
Thorington/Howell		−1.16	0.252
EF at Near			
Cover Test/Howell		5.55	<0.001
Cover Test/Thorington		4.67	<0.001
Thorington/Howell		4.76	<0.001

XF, exophoria; *p*-value for parametric statistics. Based on estimated marginal. Adjustment for multiple comparison: Bonferroni. EF, esophoria; *p*_np-value for non-parametric statistics.

**Table 3:** Predictive models for the degree of stereopsis for each phoria test used at near fixation and the break of NPC using the regression analysis. Standardized beta-value, *F*-value, *p*-value, adjusted *R*-square, *R*-value, *t*-value, and Confidence Intervals are presented for better understanding of the relationship between the analyzed variables.

STEREOPSIS							
	Standardized Beta-Value	t-Value	p-Value	R-Value	Adjusted R Square	F-Value	95% CI L–U
Break of NPC	0.46	7.58	<0.001	0.46	0.208	57.39	0.87–1.48
XF Near Cover Test	0.39	5.87	<0.001	0.40	0.159	34.95	0.85–1.7
XF Near Howell	0.44	6.46	<0.001	0.43	0.189	41.78	1.36–2.56
XF Near Thorington	0.27	3.76	<0.001	0.27	0.071	14.12	0.52–1.65

XF, exophoria; NPC, near point of convergence; CI (Confidence Interval); L (lower) and U (upper) bound.

**Table 4:** Illustrates the mean value and Std for the stereopsis degree and the break of NPC based on age-groups. It depicts how age can affect the near point of convergence and the stereopsis level of our participants. The *p* and *F*-values are represented to show the differences among them and their statistical significance.

Group	Stereopsis Mean ± Std	Break Mean ± Std	Compared Groups	*p*-Value Stereo/Break		*F*-Value Stereo/Break	*p*-Value Stereo/Break
1	40.49 ± 16.46	6.85 ± 6.84	1/2	<0.001	0.040	11.63/4.99	<0.001/0.008
2	29.57 ± 13.62	4.58 ± 5.39	1/3	0.002	0.912		
3	30.57 ± 14.94	7.32 ± 5.55	2/3	0.919	0.021		

Stereo; stereopsis degree; Break, break value of NPC. First group (5.06 to 8.00); second group (8.01 to 11.00); third group (11.01 to 17.11).

**Table 5:** Illustrates the mean value and Std for the stereopsis degree, based on the break value. These results explain how the near point of convergence can impact the stereopsis level of our participants. The *p* and *F*-values are represented to show the differences among them and their statistical significance.

Group	Stereopsis Mean ± Std	Compared Groups	*p*-Value	*F*-Value	*p*-Value
1	28.59 ± 12.56	1/2	0.509	30.14	<0.001
2	31.06 ± 12.31	1/3	<0.001		
3	47.29 ± 18.01	2/3	<0.001		

First group, break from 0–5 cm; second group, break from 6–10 cm; third group, break from 11–25 cm.

**Table 6:** Illustrates the mean value and Std for the exophoria state at far and neardistance, as a dependent variable of the break value of NPC. The obtained data show how the near point of convergence can relate to the amount of exophoria at near fixation. The *p* and *F*-values are represented to show the differences among them and their statistical significance.

Group	XF at Far Mean ± Std	XF at Near Mean ± Std	Compared Groups	*p*-Value XF F/N	*F*-Value XF F/N	*p*-Value XF F/N
1	1.71 ± 2.41	9.40 ± 4.38	1/2	0.989/0.618	7.99/20.68	<0.001
2	1.66 ± 2.28	10.10 ± 3.88	1/3	0.001/<0.001		
3	3.44 ± 2.89	14.50 ± 4.67	2/3	0.003/<0.001		

XF, exophoria; F (far); N (near). First group, break from 0–5 cm; second group, break from 6–10 cm; third group, break from 11–25 cm.

## Data Availability

The data presented in this study are available on request from the corresponding author. The data are not publicly available due to confidentiality.

## Abbreviations

The following abbreviations are used in this manuscript:

CT	Cover test
CI	confidence interval
EF	esophoria
F	far
H	Howell test
N	near
U	upper
L	lower
NPC	near point of convergence
OD	oculus dexter
OS	oculus sinister
Std	standard deviation.
TH	Thorington test
*t*-value	*t*-value of Paired Samples *t*-test for XF
VA	visual acuity
XF	exophoria
*z*-value	*z*-value of Wilcoxon Test for EFk
*p*-value	*p*-value for parametric statistics
*p*_np-value	p_np-value for non-parametric statistics
